# A Turn-Off Fluorescent Biomimetic Sensor Based on a Molecularly Imprinted Polymer-Coated Amino-Functionalized Zirconium (IV) Metal–Organic Framework for the Ultrasensitive and Selective Detection of Trace Oxytetracycline in Milk

**DOI:** 10.3390/foods12112255

**Published:** 2023-06-03

**Authors:** Xiaohui Wang, Chang Liu, Yichuan Cao, Lin Cai, Haiyang Wang, Guozhen Fang

**Affiliations:** 1 State Key Laboratory of Food Nutrition and Safety, Tianjin University of Science and Technology, Tianjin 300457, China; xhw2022tjin@163.com (X.W.);; 2 School of Food Science, Henan Institute of Science and Technology, Xinxiang 453003, China

**Keywords:** turn-off fluorescent biomimetic sensor, amino-functionalized zirconium (IV) metal–organic framework, molecularly imprinted polymer, oxytetracycline

## Abstract

Developing sensitive and effective methods to monitor oxytetracycline residues in food is of great significance for maintaining public health. Herein, a fluorescent sensor (NH_2_-UIO-66 (Zr)@MIP) based on a molecularly imprinted polymer-coated amino-functionalized zirconium (IV) metal–organic framework was successfully constructed and first used for the ultrasensitive determination of oxytetracycline. NH_2_-UIO-66 (Zr), with a maximum emission wavelength of 455 nm under 350 nm excitation, was prepared using a microwave-assisted heating method. The NH_2_-UIO-66 (Zr)@MIP sensor with specific recognition sites for oxytetracycline was then acquired by modifying a molecularly imprinted polymer on the surface of NH_2_-UIO-66 (Zr). The introduction of NH_2_-UIO-66 (Zr) as both a signal tag and supporter can strengthen the sensitivity of the fluorescence sensor. Thanks to the combination of the unique characteristics of the molecularly imprinted polymer and NH_2_-UIO-66 (Zr), the prepared sensor not only exhibited a sensitive fluorescence response, specific identification capabilities and a high selectivity for oxytetracycline, but also showed good fluorescence stability, satisfactory precision and reproducibility. The fabricated sensor displayed a fluorescent linear quenching in the OTC concentration range of 0.05–40 μg mL^−1^, with a detection limit of 0.012 μg mL^−1^. More importantly, the fluorescence sensor was finally applied for the detection of oxytetracycline in milk, and the results were comparable to those obtained using the HPLC approach. Hence, the NH_2_-UIO-66 (Zr)@MIP sensor possesses great application potential for the accurate evaluation of trace oxytetracycline in dairy products.

## 1. Introduction

Luminescent metal organic frameworks (LMOFs), as rapidly developing novel-type organic–inorganic porous crystal materials, have displayed wide application potential in sensing fields owing to their high specific surface area and porosity, intrinsic luminescence properties, as well as structure multiplicity [1,2]. Among them, NH_2_-UIO-66 (Zr) is a kind of stable zirconium-based metal–organic framework (Zr-MOF), and has been developed to construct fluorescent sensors in recent years on account of its excellent fluorescence (FL) characteristics, chemical stability and large surface area [3,4,5,6]. For instance, Zhu et al. fabricated a NH_2_-UIO-66 (Zr)-based sensor for the sensitive FL detection of the fluoride anion in water medium [6]. NH_2_-UIO-66 (Zr) was successfully utilized as a fluorescent probe for sensitively detecting the phosphate anion in aqueous media via the FL enhancement effect [7]. Wang et al. designed a novel ratio FL probe based on fluorescent Zr-MOF for the highly sensitive determination of dopamine and reduced glutathione [5]. Wang et al. stated clearly that the NH_2_-UIO-66 fluorescent sensor was triumphantly prepared and applied for the FL sensing of tetracyclines in milk [8]. Although these NH_2_-UIO-66 (Zr)-based optical sensors showed gratifying FL sensing performances, there were still some limitations, such as a relatively low selectivity for target analytes and a weak anti-interference capability in elaborate actual samples. As a result, the establishment of LMOF-based sensors with specific recognition abilities and high selectivity is a vital method by which to achieve the accurate evaluation of target analytes in real samples.

Molecular imprinting technology (MIT) is a promising molecular simulation recognition technique that mimics the specific antigen–antibody or enzyme–substrate binding processes, and has attracted great attention in the past few decades. The formed molecularly imprinted polymer (MIP) not only possesses the ability to specifically recognize the detected molecules, but also has a high adsorption efficiency and strong stability, which makes it widely used in various fields (such as catalysis, separation, drug delivery, gas sensing, chemical and biological sensing) [9,10,11,12]. In addition, MIP has been applied in optical sensors to enhance the selectivity of sensors. To date, some research has been conducting regarding the combination of NH_2_-UIO-66 (Zr) with MIP in order to design fluorescent sensors, in which the introduction of MIP can promote the generation of customized binding sites complementary to the target analytes in terms of size, shape and functional group, thus improving the selectivity of the obtained sensor. For example, a MOF@MIP FL sensor (NH_2_-UIO-66@MIP), synthetized by encapsulating NH_2_-UIO-66 into the MIP, was successfully exploited for the ultrasensitive and highly selective detection of melatonin in juice [13]. A LMOF-based sensor (NH_2_-UIO-66/MIP) was constructed to selectively and effectively detect trace levels of 4-nitrophenol in drinkable water, as well as in environmental water [14]. A turn-on FL sensor (MIP/NH_2_-UIO-66) was made via decorating MIP on Zr-LMOF and was employed for the FL sensing of chloramphenicol in animal-derived food [15]. Therefore, the integration of LMOFs and MIP in order to construct novel fluorescent sensors can supply an efficacious means by which to perform food safety supervision, such as monitoring the residual trace antibiotics.

For many decades, the abuse of antibiotics has been regarded as a global concern because antibiotic residues may constitute a threat to human health [16,17]. Oxytetracycline (OTC), a tetracycline antibiotic, has been extensively utilized in the remedy of infectious diseases induced by pathogenic microorganisms due to its broad-spectrum antimicrobial activity, effectiveness and low cost [18]. Nevertheless, the overuse of OTC will lead to its accumulation in the environment and foodstuffs, thus causing a series of potential hazards, such as anaphylactic reactions, kidney and liver damage, decreased human immune function, as well as the emergence of antibiotic-resistant bacteria [19,20,21]. To guarantee consumer safety, the European Union (EU) and China have dictated the maximum OTC residue limits (MRL) in milk products, which are 225 nM and 100 μg kg^−1^, respectively [22,23,24]. At present, plentiful analytical techniques, including capillary electrophoresis (CE) [25], liquid chromatography–tandem mass spectrometry (LC–MS/MS) [26], enzyme-linked immunosorbent assay (ELISA) [27], high-performance liquid chromatography (HPLC) [28], fluorometric assay [29] and so on, have been employed to quantitatively determine OTC. In these methods, FL sensing approaches have shown considerable superiorities over traditional analysis methods, such as a convenience of operation, an inexpensive cost, high sensibility and a rapid response, which are more appropriate for the FL sensing detection of OTC [30,31,32]. Hence, taking account of the current status of OTC, it is still indispensable to construct a new, reliable and efficient FL sensing method with which to monitor OTC in food. In view of the above research and analysis, the FL sensing strategy based on the incorporation of LMOFs and MIP has potential regarding its ability to offer a sensitive and efficacious technique with which to determine OTC.

Although some FL sensors that integrate the merits of LMOFs and MIP have been designed [13,14,15,33,34,35], hitherto, there has been no report on a fluorescent sensor based on MIP-coated NH_2_-UIO-66 (Zr) for the selective and ultrasensitive FL monitoring of OTC. Consequently, in this work, a turn-off FL sensor (expressed as NH_2_-UIO-66 (Zr)@MIP) that combines a large surface area and the unique fluorescence properties of NH_2_-UIO-66 (Zr), as well as the high specificity of the imprinted layer, was developed for an assay of OTC using the inner filter effect (IFE). Herein, the NH_2_-UIO-66 (Zr) with an intense blue FL emission, as a signal tag and substrate material, was prepared via a simple microwave-assisted heating process and then covered with a molecularly imprinted layer. After removing the OTC template molecule, the NH_2_-UIO-66 (Zr)@MIP sensor with custom-made binding sites for the OTC was obtained. The modification of the MIP granted the sensor high selectivity, adsorption affinity and binding efficiency to OTC. Meanwhile, NH_2_-UIO-66 (Zr), as a luminous center and a carrier, endowed the proposed sensor with a desirable FL signal output and response sensitivity. Furthermore, its relatively uniform morphology, good optical properties, superior FL sensitivity, outstanding adsorption and binding performance, as well as its excellent specificity, were demonstrated via different characterizations. The established sensor was successfully used in the highly sensitive and selective determination of OTC in milk samples, confirming its practical applicability.

## 2. Materials and Methods

### 2.1. Materials and Apparatuses

2-aminoterephthalic acid (ATA, 98%), tetracycline (TET, ≥98%), doxycycline (DOX, ≥98%), oxytetracycline (OTC, ≥98%), chlortetracycline (CTC, ≥98%), chloramphenicol (CAP, 98%), tyrosine (Tyr), histidine (His), serine (Ser), phenylalanine (Phe), arginine (Arg) and cysteine (Cys) were provided by Shanghai Macklin Biochemical Co., Ltd. (Shanghai, China). Zirconium chloride (ZrCl_4_, 99%) was purchased from Beijing Yinuokai Technology Co., Ltd. (Beijing, China). Methacrylic acid (MAA, 99%), 2,2-azobisisobutyronitrile (AIBN, 99%) and ethylene glycol dimethacrylate (EGDMA, 98%) were obtained from TCI Development Co., Ltd. (Shanghai, China). N, N-dimethylformamide (DMF), ethanol, glucose, sucrose, MnCl_2_·4H_2_O, MgCl_2_·6H_2_O, Zn(NO_3_)_2_·6H_2_O, CaCl_2_, NaCl and KCl were supplied by Sinopharm Chemical Reagent Co., Ltd. (Shanghai, China). All reagents and chemicals used in this work were of at least analytical grade. The apparatuses utilized in this study are described in detail in the supporting information.

### 2.2. The Synthesis of NH_2_-UIO-66 (Zr)

The preparation process of NH_2_-UIO-66 (Zr) refers to the microwave-assisted heating assay employed in a previous study with minor changes [6]. Briefly, 669 mg of ZrCl_4_ and 500 mg of ATA were firstly dissolved in 60 mL of DMF, followed by ultrasonic treatment for 1 h. Next, the aforementioned reaction precursor solution was placed in a stainless steel Teflon-lined autoclave and heated to 120 °C for 24 h. After natural cooling, the deposition was attained via centrifugation, distributed in ethanol (30 mL), and heated at 100 °C for 30 min in a microwave synthesis reaction device. Subsequently, after dropping to ambient temperature, the obtained solid was centrifuged, and then the above microwave heating activation process was carried out again. Ultimately, the yellow product was acquired via vacuum drying (60 °C for 6 h) for later tests.

### 2.3. The Fabrication of NH_2_-UIO-66 (Zr) Imprinted Polymers

NH_2_-UIO-66 (Zr)@MIP was fabricated according to a previous study, with slight modifications [15]. In the typical procedure, NH_2_-UIO-66 (Zr) (fluorescent probe, 50 mg) and OTC (template, 0.2 mmol) were completely dispersed in 30 mL of ethanol using ultrasonic treatment. Then, after the addition of MAA (monomer, 1.2 mmol), the mixture was pre-polymerized for 50 min under continuous stirring. Subsequently, EGDMA (cross-linking agent, 2 mmol) and AIBN (initiator, 20 mg) were successively introduced into the above synthesis system and stirred for 15 h in a water bath at 65 °C. Finally, the acquired polymer was centrifuged, eluted repeatedly via water/ethanol (*v*/*v*, 1/9) to expunge OTC and dried under vacuum at 50 °C for 7 h. Meanwhile, the non-imprinting polymer (NH_2_-UIO-66 (Zr)@NIP), as a contrast material, was prepared according to the above steps in addition to not adding OTC.

### 2.4. FL Sensing Procedure

In brief, 1 mg of NH_2_-UIO-66 (Zr)@MIP or NH_2_-UIO-66 (Zr)@NIP was scattered in 4 mL of PBS buffer solution (pH = 7) with various concentrations of OTC. After incubation for 30 min, the FL determination was performed under an excitation wavelength of 350 nm and the FL intensity of the sensing system at 455 nm was obtained. The specific recognition performance was appraised by adding other antibiotics with a similar structure or possible coexisting interfering substances instead of OTC under identical experimental conditions. The measurements of each experiment were carried out in triplicate.

### 2.5. Real Samples Analysis

Fresh pure milk bought from a local supermarket (labeled as Milk 1, Milk 2, and Milk 3, according to brand difference) was used to assess the practicability of the proposed FL method based on NH_2_-UIO-66 (Zr)@MIP, by spiking the proper concentration of OTC. Three parallel experiments were conducted on the milk samples of each concentration.

The spiked milk samples containing specific OTC concentrations were first prepared and placed overnight. Afterwards, the preprocessing of the samples was executed according to a previous study [36]. In brief, 1 mL milk samples with various OTC concentrations (0 μg mL^−1^, 1 μg mL^−1^, 15 μg mL^−1^ and 25 μg mL^−1^) were vortically mingled with 4 mL of acetonitrile for 5 min. Subsequently, the mixture was centrifuged and the resulting supernatant was filtrated using a 0.22 μm microfilter to expunge protein precipitates. Finally, the above samples were dried using nitrogen and re-dispersed in 1 mL of PBS buffer solution (pH = 7) for FL determination.

### 2.6. HPLC Analysis

With regard to HPLC determination, the extraction, purification and analysis procedures of milk samples were carried out according to Chinese National Standards (GB/T 22990-2008) [37], which were provided in the supporting information.

## 3. Results and Discussion

### 3.1. Synthesis of NH_2_-UIO-66 (Zr) and NH_2_-UIO-66 (Zr)@MIP

**Scheme 1:**
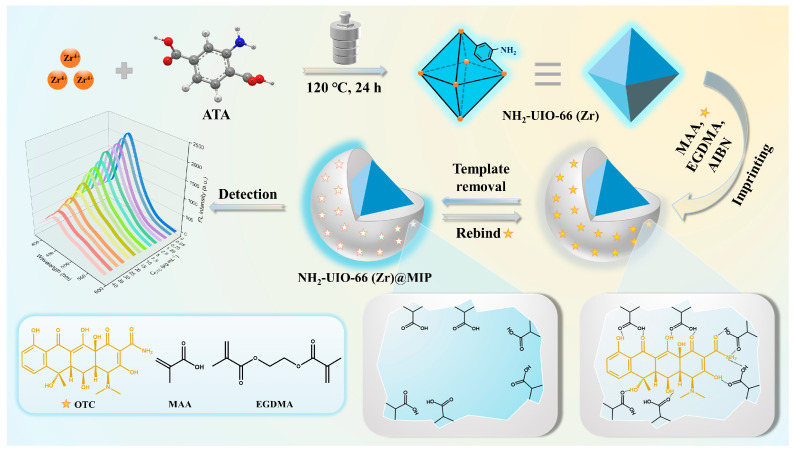
Schematic diagram of the preparation of NH_2_-UIO-66 (Zr) and NH_2_-UIO-66 (Zr)@MIP.

As portrayed in Scheme 1, NH_2_-UIO-66 (Zr) with a bright blue fluorescence and large surface area was first fabricated via a self-assembly strategy using Zr^4+^ and ATA as precursors. Subsequently, a new biomimetic FL sensor based on MIP-coated NH_2_-UIO-66 (Zr) was prepared using the one-step aggregation method. The polymerization reaction was thermally initiated and completed in a water bath in the presence of OTC (template), NH_2_-UIO-66 (Zr) (fluorophore and supporter), MAA (functional monomer), EGDMA (cross-linker) and AIBN (initiator). During this process, OTC molecules and functional monomers were combined by hydrogen bonds. After eliminating the imprinted template OTC by using a suitable eluent, the NH_2_-UIO-66 (Zr)@MIP sensor with specific recognition cavities fitting the shape and functional group of OTC was finally achieved in order to selectively monitor the OTC. The existence of NH_2_-UIO-66 (Zr) as both a signal transducer and carrier granted the proposed sensor outstanding FL characteristics and good response sensitivity. The introduction of a molecularly imprinted layer not only preserved the sensitive FL signal of NH_2_-UIO-66 (Zr), but also endowed the obtained sensor with the ability to specifically recognize OTC. Consequently, the constructed NH_2_-UIO-66 (Zr)@MIP sensor integrated these superiorities of LMOFs and MIP. Moreover, in order to obtain NH_2_-UIO-66 (Zr)@MIP with an excellent fluorescent response and the ability to bind well to the OTC, the synthesis conditions, including the additive level of fluorophore, the molar ratio of OTC, MAA and EGDMA, and the polymerization time, were optimized (Tables S1–S3).

### 3.2. Characterization

**Figure 1:**
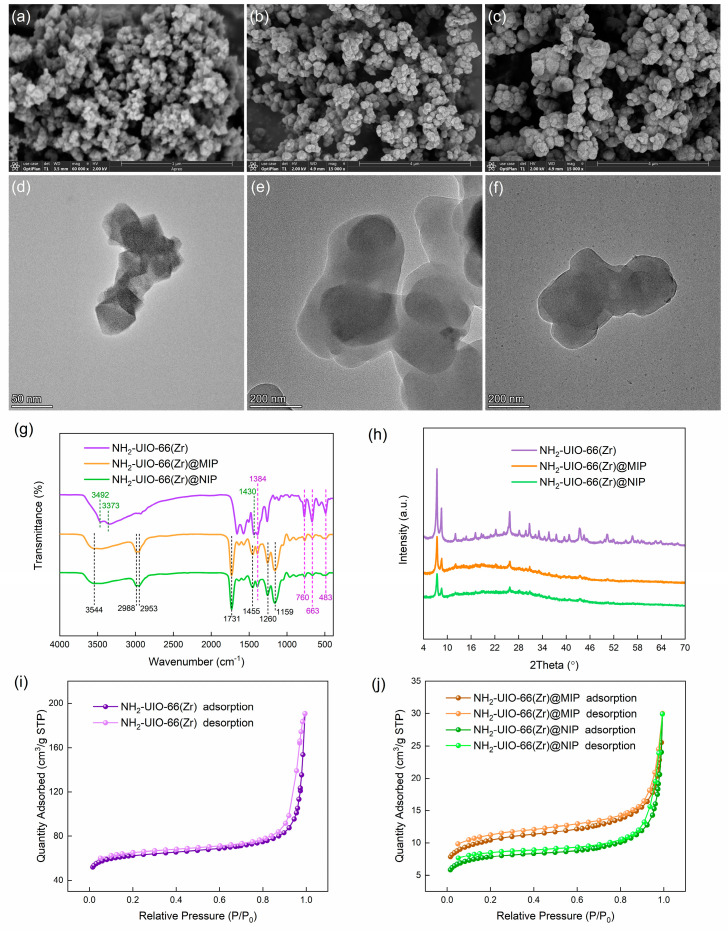
SEM images of (**a**) NH_2_-UIO-66 (Zr), (**b**) NH_2_-UIO-66 (Zr)@MIP and (**c**) NH_2_-UIO-66 (Zr)@NIP. TEM images of (**d**) NH_2_-UIO-66 (Zr), (**e**) NH_2_-UIO-66 (Zr)@MIP and (**f**) NH_2_-UIO-66 (Zr)@NIP. (**g**) FT–IR spectra and (**h**) XRD patterns of NH_2_-UIO-66 (Zr), NH_2_-UIO-66 (Zr)@MIP and NH_2_-UIO-66 (Zr)@NIP. (**i**) Nitrogen adsorption and desorption curves of NH_2_-UIO-66 (Zr). (**j**) Nitrogen adsorption and desorption curves of NH_2_-UIO-66 (Zr)@MIP and NH_2_-UIO-66 (Zr)@NIP.

The surface morphologies and structures of the fabricated NH_2_-UIO-66 (Zr), NH_2_-UIO-66 (Zr)@MIP and NH_2_-UIO-66 (Zr)@NIP were observed via SEM and TEM. SEM (Figure 1a), TEM (Figure 1d) and diameter distribution images (Figure S1) revealed that NH_2_-UIO-66 (Zr) presented a uniform and smooth octahedron-like appearance, with a mean size of nearly 50 nm [38,39]. The elemental composition of NH_2_-UIO-66 (Zr) was also explored via the energy-dispersive spectrometer (EDS) spectrum (Figure S2), confirming the coexistence of C, N, O and Zr elements in NH_2_-UIO-66 (Zr). These results demonstrated the successful synthesis of NH_2_-UIO-66 (Zr). As displayed in Figure 1b,c, the obtained polymers (NH_2_-UIO-66 (Zr)@MIP and NH_2_-UIO-66 (Zr)@NIP) appeared nearly spherical, with a particle size of approximately 250 nm (Figure S3), and their surface was rougher than that of NH_2_-UIO-66 (Zr) due to the introduction of the molecular imprinting layer. The TEM images (Figure 1e,f) showed that the polymers basically maintained the frame of NH_2_-UIO-66 (Zr) and possessed an archetypal core-shell structure, which evidenced that the polymers were successfully formed. In addition, the element mapping image of NH_2_-UIO-66 (Zr)@MIP (Figure S4) showed the homogeneous distribution of the main characteristic elements in the polymer, preliminarily verifying the triumphant construction of the NH_2_-UIO-66 (Zr)@MIP sensor.

The FT–IR spectra were recorded in order to analyze the surface chemistry of the synthetic materials. As presented in Figure 1g, in the spectrum of NH_2_-UIO-66 (Zr), the characteristic peaks at 3492 and 3373 cm^−1^ can be attributed to the antisymmetric and symmetric stretching modes of -NH_2_, respectively [8,40]. The absorption bands in the range of 1400–1500 cm^−1^ originated from a C=C stretching vibration in the benzene ring of the ATA ligand [14,15]. The stretching absorption peaks of O–Zr–O (760 and 663 cm^−1^), Zr–O (483 cm^−1^) and -OCO (1430 and 1384 cm^−1^) were also observed, indicating that the dehydroxylation phase in NH_2_-UIO-66 (Zr) was favorably formed thanks to the coordination of Zr^4+^ with ATA [6,41]. For NH_2_-UIO-66 (Zr)@NIP, the absorption peaks at 3544 cm^−1^ (-OH), 2988 and 2953 cm^−1^ (C=H), 1731 cm^−1^ (C=O), 1455 cm^−1^ (C=C), and 1260 and 1159 cm^−1^ (C–O–C) symbolized the presence of MAA (functional monomer) and EGDMA (cross-linker). Meanwhile, the Zr–O vibration peaks (760, 663 and 483 cm^−1^) corresponding to the NH_2_-UIO-66 (Zr) appeared in NH_2_-UIO-66 (Zr)@NIP, which demonstrated the successful preparation of the non-imprinted polymer doped with NH_2_-UIO-66 (Zr) [13,14,42]. Additionally, the position and intensity of the characteristic peaks in the NH_2_-UIO-66 (Zr)@MIP spectrum were very similar to those in the NH_2_-UIO-66 (Zr)@NIP spectrum, implying that NH_2_-UIO-66 (Zr)@MIP was triumphantly developed.

To analyze the crystallographic structure of the synthesized materials, XRD data were collected and are depicted in Figure 1h. The characteristic diffraction peaks (2θ = 7.37°, 8.6°, 12.05°, 17.06°, 22.17° and 25.72°) of NH_2_-UIO-66 (Zr) highly coincided with those reported in the literature, which showed that NH_2_-UIO-66 (Zr) with good crystallinity was triumphantly fabricated [13,14,43]. Notably, in the XRD patterns of NH_2_-UIO-66 (Zr)@MIP and NH_2_-UIO-66 (Zr)@NIP, the characteristic absorption peaks associated with NH_2_-UIO-66 (Zr) became unapparent, owing to the decoration of the surface molecularly imprinted shell, which helped to prove the successful preparation of the polymers [43,44].

In order to further investigate the surface characteristics of the resulting materials, the N_2_ adsorption/desorption isotherms of NH_2_-UIO-66 (Zr) and its imprinted polymer were evaluated, as shown in Figure 1i,j. The corresponding specific surface areas of NH_2_-UIO-66 (Zr), NH_2_-UIO-66 (Zr)@MIP and NH_2_-UIO-66 (Zr)@NIP, obtained via the Brunauer–Emmett–Teller (BET) analysis method, were 219 m^2^ g^−1^, 37 m^2^ g^−1^ and 28 m^2^ g^−1^, respectively. Compared to NH_2_-UIO-66 (Zr), the surface areas of NH_2_-UIO-66 (Zr)@MIP and NH_2_-UIO-66 (Zr)@NIP were clearly reduced after coating the imprinted layer [14,35,45]. Meanwhile, there was a slight increase in the surface area of NH_2_-UIO-66 (Zr)@MIP in comparison to that of NH_2_-UIO-66 (Zr)@NIP, probably due to the existence of imprinted cavities after eliminating the template [46].

The above-mentioned data signified that the FL sensor (NH_2_-UIO-66 (Zr)@MIP) was triumphantly fabricated via combining the molecularly imprinted polymer with NH_2_-UIO-66 (Zr).

### 3.3. Optical and Adsorption Properties of NH_2_-UIO-66 (Zr)@MIP

**Figure 2:**
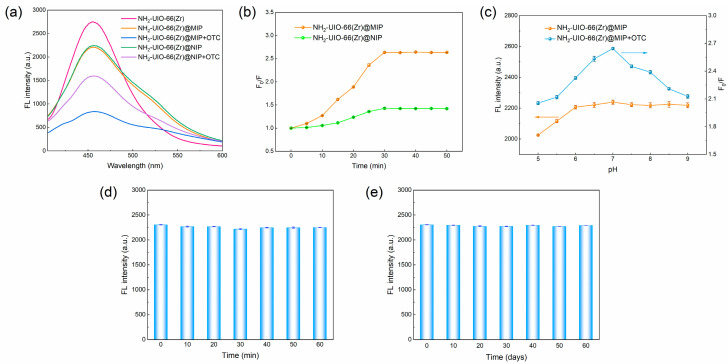
(**a**) FL emission spectra of NH_2_-UIO-66 (Zr), NH_2_-UIO-66 (Zr)@MIP, NH_2_-UIO-66 (Zr)@MIP+OTC, NH_2_-UIO-66 (Zr)@NIP and NH_2_-UIO-66 (Zr)@NIP+OTC. (**b**) FL response of NH_2_-UIO-66 (Zr)@MIP and NH_2_-UIO-66 (Zr)@NIP towards OTC at different incubation times. (**c**) Effects of pH values (5–9) on the FL intensity of NH_2_-UIO-66 (Zr)@MIP, as well as the FL response between NH_2_-UIO-66 (Zr)@MIP and OTC. The influence of (**d**) irradiation time at 350 nm and (**e**) storage period on the FL intensity of NH_2_-UIO-66 (Zr)@MIP.

The luminescence performance of the developed sensor was firstly studied through FL spectra. As displayed in Figure 2a, NH_2_-UIO-66 (Zr)@MIP showed slightly lower FL intensity than NH_2_-UIO-66 (Zr) on account of decorating the MIP layer. However, NH_2_-UIO-66 (Zr)@MIP exhibited an intense symmetric FL emission peak corresponding to NH_2_-UIO-66 (Zr) at 455 nm under 350 nm excitation, which implied that the modification of the surface imprinting layer had no remarkable impact on the FL performance of NH_2_-UIO-66 (Zr). As a consequence, the synthesized sensor maintained the unique luminous property of NH_2_-UIO-66 (Zr).

FL experiments measuring the response of the polymers (NH_2_-UIO-66 (Zr)@MIP and NH_2_-UIO-66 (Zr)@NIP) to OTC were then conducted to explore the practicability of the established sensor (Figure 2a). Clearly, when OTC existed in the detection system, the FL intensity of the polymers at 455 nm was effectively decreased; meanwhile, NH_2_-UIO-66 (Zr)@MIP presented a more conspicuous FL change than NH_2_-UIO-66 (Zr)@NIP, owing to its better binding capacity and specificity towards OTC. After eliminating OTC, the fluorescence of the polymers was restored and the FL spectrum of NH_2_-UIO-66 (Zr)@MIP was basically consistent with that of NH_2_-UIO-66 (Zr)@NIP, which indicated that the fabricated NH_2_-UIO-66 (Zr)@MIP sensor was viable for the determination of OTC.

Adsorptive kinetics tests of the as-prepared polymers were performed to investigate the adsorption performance of the sensor. As shown in Figure 2b, the FL response (*F*_0_/*F*) of NH_2_-UIO-66 (Zr)@MIP and NH_2_-UIO-66 (Zr)@NIP towards the target OTC at different incubation times was recorded. Under the continuously increasing reaction time, the *F*_0_/*F* value of NH_2_-UIO-66 (Zr)@MIP increased quickly at first, reached the maximum at 30 min, and then tended to remain unchanged, which demonstrated that the detection system achieved adsorption equilibrium within 30 min. Therefore, 30 min was considered to be the optimal reacting time in consequent trials. The variation in the trend observed with regard to the FL response of NH_2_-UIO-66 (Zr)@NIP was analogous to NH_2_-UIO-66 (Zr)@MIP. Nevertheless, NH_2_-UIO-66 (Zr)@MIP possessed a higher FL response and adsorption efficiency than NH_2_-UIO-66 (Zr)@NIP at the identical reaction time, indicating that the generation of specific identification imprinted sites enhanced the binding affinity of NH_2_-UIO-66 (Zr)@MIP towards OTC.

Next, the influences of the pH (5–9) on the fluorescence intensity of NH_2_-UIO-66 (Zr)@MIP and the FL response of NH_2_-UIO-66 (Zr)@MIP towards OTC were studied in the case of not adding and adding OTC, respectively. As illustrated in Figure 2c, with the pH changing from 5 to 9, the FL intensity of NH_2_-UIO-66 (Zr)@MIP first increased, then remained basically stable, and the highest FL intensity was apparently observed at pH = 7. In addition, the maximum FL response value (*F*_0_/*F*) was concurrently obtained at pH = 7, proving that NH_2_-UIO-66 (Zr)@MIP possessed the strongest adsorption binding efficiency to OTC. Thus, pH = 7 was chosen as the best pH for the sensing system in subsequent FL detection experiments.

Finally, the fluorescent stability of the obtained sensor was estimated by measuring the FL intensity at 455 nm of NH_2_-UIO-66 (Zr)@MIP under diverse ultraviolet (UV) light radiation times and storing times. As shown in Figure 2d, NH_2_-UIO-66 (Zr)@MIP exhibited an unapparent FL change after UV irradiation at 350 nm for 60 min, signifying the exceptional photo-bleaching resistance of the proposed NH_2_-UIO-66 (Zr)@MIP-based sensor. At the same time, the FL intensity of NH_2_-UIO-66 (Zr)@MIP remained steady within 60 days (Figure 2e), which indicated the superior storage stability of the resulting sensor.

The above experimental results confirm that the NH_2_-UIO-66 (Zr)@MIP-based sensor had good optical properties and a good adsorption performance.

### 3.4. Construction of FL Detection System for OTC

**Figure 3:**
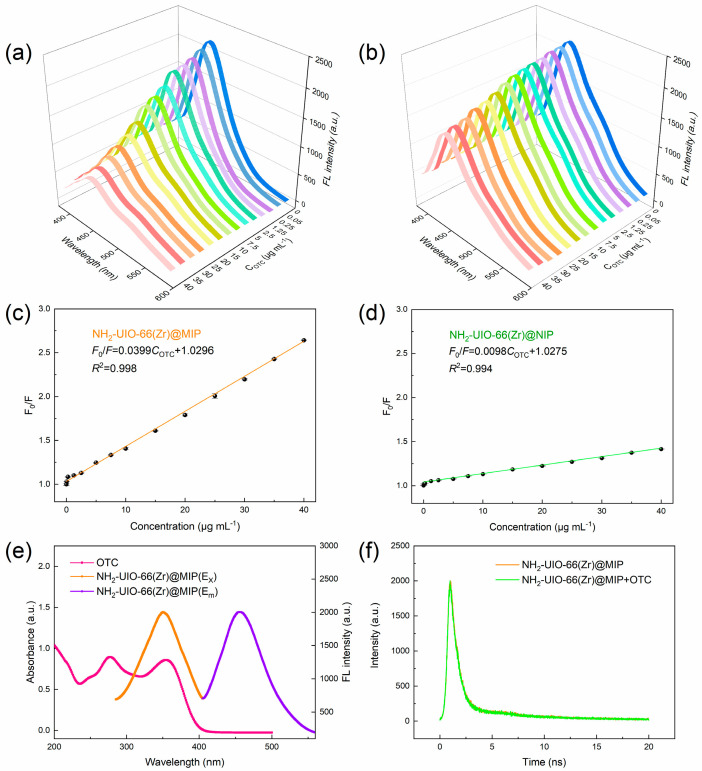
FL spectra of (**a**) NH_2_-UIO-66 (Zr)@MIP and (**b**) NH_2_-UIO-66 (Zr)@NIP under different concentrations of OTC (excited at 350 nm). The Stern–Volmer plots for NH_2_-UIO-66 (Zr)@MIP (**c**) and NH_2_-UIO-66 (Zr)@NIP (**d**). (**e**) The UV absorption spectrum of OTC and the FL spectra of NH_2_-UIO-66 (Zr)@MIP (Ex, FL excitation spectrum; Em, FL emission spectrum). (**f**) FL emission decay curves of NH_2_-UIO-66 (Zr)@MIP alone and NH_2_-UIO-66 (Zr)@MIP with 40 μg mL^−1^ concentrations of OTC.

Under the above optimum sensing conditions, the concentration responses of the as-synthesized FL polymers for OTC were assessed by collecting and analyzing the FL spectra of NH_2_-UIO-66 (Zr)@MIP and NH_2_-UIO-66 (Zr)@NIP after the addition of different levels of OTC. As portrayed in Figure 3a, by increasing the OTC concentration from 0.05 to 40 μg mL^−1^, the FL intensity of NH_2_-UIO-66 (Zr)@MIP was progressively quenched. Clearly, NH_2_-UIO-66 (Zr)@NIP showed an analogous FL change trend towards NH_2_-UIO-66 (Zr)@MIP. Nevertheless, compared with NH_2_-UIO-66 (Zr)@NIP, NH_2_-UIO-66 (Zr)@MIP presented a more noticeable FL quenching response when the same concentration of OTC was added (Figure 3b), which was the result of the presence of molecularly imprinted sites matching OTC in NH_2_-UIO-66 (Zr)@MIP. Furthermore, the relationship between the FL quenching signal (*F*_0_/*F*) and the concentration of OTC can be described via the Stern–Volmer equation [14,47]:

*F*_0_/*F* = 1 + *K*_SV_*C*_OTC_

where *C*_OTC_ and *K_SV_* are the concentration of OTC and the FL quenching constant, respectively. *F*_0_ and *F* represent the FL intensity of the sensing system before and after adding OTC. The imprinting factor (*IF*), set as the specific value of *K*_SV, NH2-UIO-66 (Zr)@MIP_ to *K*_SV, NH2-UIO-66 (Zr)@NIP_, was used as a key index to estimate the adsorption binding capacity of the NH_2_-UIO-66 (Zr)@MIP sensor towards OTC.

Figure 3c shows that there was a linear dependence between the FL quenching signal of NH_2_-UIO-66 (Zr)@MIP and the OTC concentration (0.05–40 μg mL^−1^). The limit of detection (LOD) of 0.012 μg mL^−1^ was achieved on the basis of the standard 3*S_b_*/*slope* (*S_b_* = 1.63 × 10^−4^, was the standard deviation of blank measurements, *n* = 9) [22,48]. Plainly, NH_2_-UIO-66 (Zr)@MIP had a more notable FL response change than NH_2_-UIO-66 (Zr)@NIP at an identical OTC dose, which emphasized the outstanding selectivity and adsorptive efficiency of NH_2_-UIO-66 (Zr)@MIP towards OTC. Simultaneously, the good specific recognition capacity of NH_2_-UIO-66 (Zr)@MIP was also authenticated by comparing the slopes of the corresponding standard linear equations of NH_2_-UIO-66 (Zr)@MIP (*F*_0_/*F* = 0.0399*C*_OTC_ + 1.0296, *R^2^* = 0.998) and NH_2_-UIO-66 (Zr)@NIP (*F*_0_/*F* = 0.0098*C*_OTC_ + 1.0275, *R^2^* = 0.994) (Figure 3c,d). Moreover, the maximum *IF* value was calculated as 3.95, further revealing that the NH_2_-UIO-66 (Zr)@MIP sensor had a superior selective identification capacity and superior adsorption affinity for OTC.

The potential FL sensing mechanism of the NH_2_-UIO-66 (Zr)@MIP sensor towards the OTC molecule was investigated and discussed. The FL spectrum of NH_2_-UIO-66 (Zr)@MIP and the UV absorption spectrum of OTC are illustrated in Figure 3e. Evidently, the FL emission spectrum of NH_2_-UIO-66 (Zr)@MIP almost did not overlap with the UV absorption spectrum of OTC, but there was a partial and effective overlap between the excitation spectrum of NH_2_-UIO-66 (Zr)@MIP and the UV absorption band of OTC. Therefore, the possible mechanism was initially determined as the inner filter effect (IFE), rather than FL resonance energy transfer (FRET) [37,49]. To be specific, the FL lifetime of the fluorescent material decreases in the FRET process, while it remains invariant if IFE occurs [50,51]. Accordingly, to further elucidate the FL quenching phenomenon, the FL lifetime of NH_2_-UIO-66 (Zr)@MIP before and after OTC adsorption was analyzed. The FL emission decay curves are presented in Figure 3f. It is noteworthy that the FL lifetime of NH_2_-UIO-66 (Zr)@MIP showed a slight variation from 3.61 ns to 3.48 ns after OTC treatment, further demonstrating that the OTC-initiated FL quenching behavior was mainly caused by IFE.

### 3.5. Selectivity and Specificity Analysis

**Figure 4:**
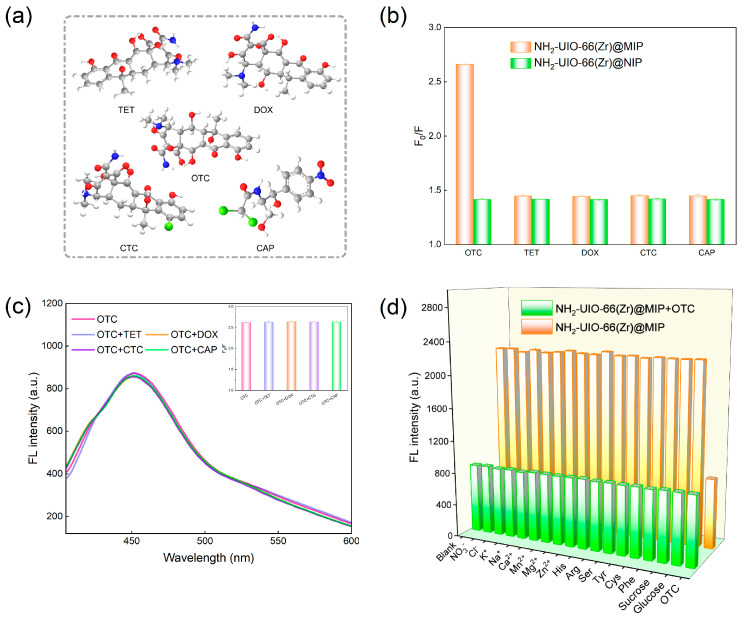
(**a**) The chemical structural formulas of OTC, TET, DOX, CTC and CAP. (**b**) The FL response (*F_0_*/*F*) of NH_2_-UIO-66 (Zr)@MIP and NH_2_-UIO-66 (Zr)@NIP to 40 μg mL^−1^ of OTC, TET, DOX, CTC and CAP. (**c**) The influence of TET, DOX, CTC and CAP (80 μg mL^−1^) on the combination of OTC (40 μg mL^−1^) and NH_2_-UIO-66 (Zr)@MIP. (**d**) The influence of coexisting substances (200 μg mL^−1^) on the FL change of NH_2_-UIO-66 (Zr)@MIP in the absence or presence of OTC (40 μg mL^−1^).

The selectivity of the constructed FL sensing system was assessed by recording and contrasting the FL changes in the polymers to OTC and its structural analogues (TET, DOX, CTC and CAP). The chemical structures of these mentioned antibiotics, which are first portrayed in Figure 4a, enable the specific recognition behavior of the NH_2_-UIO-66 (Zr)@MIP-based sensor towards the OTC molecule to be better understood. As presented in Figure 4b, the influence of OTC on the FL quenching degree of NH_2_-UIO-66 (Zr)@MIP was notably greater than that of other antibiotics with a similar structure, which was mainly owing to the formation of the special recognition cavities in NH_2_-UIO-66 (Zr)@MIP that utterly conformed the size, shape and functional group of OTC during the molecular imprinting process. Because there were no specific recognition sites corresponding to TET, DOX, CTC and CAP in NH_2_-UIO-66 (Zr)@MIP, the NH_2_-UIO-66 (Zr)@MIP sensor had a comparatively small FL response value (*F*_0_/*F*) to these analogs (Figure 4b). Therefore, it was difficult for NH_2_-UIO-66 (Zr)@MIP to selectively capture and effectively bind with these analogues in the detection process. Furthermore, compared with NH_2_-UIO-66 (Zr)@MIP, NH_2_-UIO-66 (Zr)@NIP, without specific recognition sites, generated a relatively low FL response to OTC and other analogues, which was attributed to the nonspecific adsorption between NH_2_-UIO-66 (Zr)@NIP and these analytes. The above results suggest that the introduction of highly selective imprinted sites endowed the NH_2_-UIO-66 (Zr)@MIP sensor with the ability to specifically identify OTC.

The FL response changes (*F*_0_/*F*) of NH_2_-UIO-66 (Zr)@MIP to different concentrations of structural analogues were also studied to investigate the selectivity of the established sensor. As shown in Figure S5, in the absence of OTC, the FL detection system exhibited slight differences in FL changes for different analogues, but the FL changes (*F*_0_/*F*) apparently increased after introducing OTC. In addition, there was no significant linear relationship between the concentration of the analogue and FL change (*F*_0_/*F*), which indicated that the constructed FL detection system had good selectivity for the target OTC.

In order to further assess the ability of NH_2_-UIO-66 (Zr)@MIP to specifically recognize OTC, a competitive adsorption test was conducted by adding the possible coexisting structural analogs (TET, DOX, CTC and CAP) into the mixed system containing NH_2_-UIO-66 (Zr)@MIP and OTC. As revealed in Figure 4c, NH_2_-UIO-66 (Zr)@MIP displayed tiny FL variation even though the concentration of each analogue was twice that of OTC, demonstrating that the interference caused by the presence of the coexisting analogues in the sensing system was inconspicuous. The good specificity of NH_2_-UIO-66 (Zr)@MIP towards OTC was probably due to the existence of OTC-imprinted sites generated via hydrogen bond interaction, which improved the combination efficiency of the sensor to OTC, and inhibited the nonspecific adsorption of the coexisting analogs. Thus, the obtained sensor possessed gratifying selectivity and specificity for the target OTC.

Due to the matrix complexity of the practical samples, the potential coexisting interfering substances in the real detection process, such as sugars (glucose and sucrose), ions (Cl^−^, NO_3_^−^, K^+^, Na^+^, Ca^2+^, Zn^2+^, Mg^2+^, and Mn^2+^) and common amino acids (Arg, Cys, Phe, Tyr, His and Ser), will directly influence the accuracy of the test results. Therefore, the ability of the NH_2_-UIO-66 (Zr)@MIP sensor to avoid interference was explored by introducing the above interfering analytes into the constructed FL sensing system in the presence and absence of OTC. As shown in Figure 4d, the concentration of the added interfering species was up to 200 μg mL^−1^ (5 times of OTC concentration), but the influence of these interfering substances on the FL intensity of NH_2_-UIO-66 (Zr)@MIP was minor, which was in accordance with the blank experimental group. After adding OTC, the FL of the sensing system was significantly quenched, indicating that these interfering species had a negligible effect on the FL detection of OTC. The above results indicated that the developed FL sensor exhibited excellent selectivity and specificity towards OTC, as well as superior anti-interference characteristics in a complicated environment, thus providing a feasible tool for the FL detection of OTC.

### 3.6. Real Sample Analysis

The commercial milk bought from the local supermarket was taken as a practical sample in order to analyze and authenticate the applicability of the FL sensing approach created in this study. No OTC was found in the blank milk samples when using the NH_2_-UIO-66 (Zr)@MIP method and HPLC means. Consequently, the spiking and recovery tests were performed by introducing different levels of OTC standards (0, 1, 15 and 25 μg mL^−1^) into the milk samples, and the relevant analysis results are presented in Table 1. The recovery rates of the milk samples obtained by using the established optical sensing system were between 93.56% and 98.21%, and the relative standard deviation (RSD) was <2.59%, which was comparable to the results of the HPLC method. These satisfactory data confirmed that the fabricated sensor was practicable for quantitatively detecting OTC in milk samples.

### 3.7. Accuracy Assessment of the Established Fluorescence Method

The intra-day and inter-day precisions of the established NH_2_-UIO-66 (Zr)@MIP sensing method were also appraised by detecting the spiked samples using different concentrations of OTC standards (1, 15 and 25 μg mL^−1^). As shown in Table S4, the corresponding RSD values were lower than 3.05% and 4.07% (*n* = 6), respectively, indicating that the sensor had sterling reproducibility as well as good accuracy, and was suitable for the determination of OTC in real products.

### 3.8. Approach Performance Comparison

The performance of the designed sensing assay was compared to some subsistent FL-based methods for OTC detection, the results of which are shown in Table S5. Visibly, the fluorescent sensing approach established in this study exhibited a relatively wide linear range (0.05–40 μg mL^−1^), a lower LOD (0.012 μg mL^−1^), a comparable recovery rate (93.56–98.21%) and an acceptable accuracy (RSD < 2.59%). The good performance of the NH_2_-UIO-66 (Zr)@MIP-based sensor came from the following aspects. On one hand, introducing NH_2_-UIO-66 (Zr), which has sterling FL characteristics and a large surface area, as the support improved the sensitivity of the proposed FL sensor. On the other hand, the integration of the surface-imprinted layer endowed the sensor with the ability to specifically identify and accurately absorb the target from the complicated matrix, which helped to avoid the tedious sample pretreatment process and to reduce the analysis time. Taken together, the constructed NH_2_-UIO-66 (Zr)@MIP-based sensor can be appropriately applied in order to quantitatively monitor trace OTC in a sophisticated system.

## 4. Conclusions

In summary, a novel turn-off fluorescent biomimetic sensor (NH_2_-UIO-66 (Zr)@MIP) was successfully designed based on an amino-functionalized zirconium (IV) metal–organic framework embedded in a molecularly imprinted polymer in order to selectively recognize and sensitively detect trace OTC. Because of the combination of MIP, with an excellent specific recognition ability, and NH_2_-UIO-66 (Zr), with unique fluorescent characteristics, the fabricated NH_2_-UIO-66 (Zr)MIP sensor showed exceptional sensitivity, excellent selectivity, desirable anti-interference capacity, as well as good precision during the process of detecting OTC. Additionally, the constructed FL sensing system was triumphantly employed in order to determine OTC in milk with satisfying results, displaying good practical application prospects. Therefore, it is expected that the proposed NH_2_-UIO-66 (Zr)@MIP-based sensor will provide a significant detection platform for OTC residue analysis in food safety evaluation.

## Figures and Tables

**Table 1:** Detection of OTC in milk samples using the NH_2_-UIO-66 (Zr)@MIP sensor and HPLC.

Sample	Spiked (μg mL^−1^)	NH_2_-UIO-66 (Zr)@MIP			HPLC		
		Found (μg mL^−1^)	Recovery (%)	RSD (%, *n* = 3)	Found (μg mL^−1^)	Recovery (%)	RSD (%, *n* = 3)
Milk 1	0.00	0.00	-	-	0.00	-	-
	1.00	0.95 ± 0.02	95.38 ± 2.47	2.59	0.96 ± 0.02	96.16 ± 2.50	2.60
	15.00	14.50 ± 0.29	96.65 ± 1.96	2.03	14.73 ± 0.10	98.23 ± 0.66	0.67
	25.00	24.45 ± 0.16	97.79 ± 0.65	0.67	24.54 ± 0.13	98.14 ± 0.51	0.52
Milk 2	0.00	0.00	-	-	0.00	-	-
	1.00	0.94 ± 0.02	93.56 ± 1.86	1.98	0.97 ± 0.02	96.68 ± 1.88	1.94
	15.00	14.67 ± 0.14	97.78 ± 0.91	0.93	14.78 ± 0.04	98.51 ± 0.26	0.27
	25.00	24.33 ± 0.11	97.32 ± 0.43	0.44	24.52 ± 0.12	98.10 ± 0.47	0.48
Milk 3	0.00	0.00	-	-	0.00	-	-
	1.00	0.94 ± 0.01	93.67 ± 1.00	1.07	0.96 ± 0.01	95.59 ± 1.10	1.15
	15.00	14.73 ± 0.17	98.21 ± 1.10	1.12	14.64 ± 0.01	97.62 ± 0.04	0.04
	25.00	24.41 ± 0.16	97.65 ± 0.64	0.65	24.61 ± 0.09	98.45 ± 0.35	0.35

## Data Availability

The data presented in this study are available on request from the corresponding author.

## Supplementary Materials

The following supporting information can be downloaded at: https://www.mdpi.com/article/10.3390/foods12112255/s1, Refs. [52,53,54,55,56], Instruments, The detection conditions and sample analysis procedures for HPLC method, Optimization of synthesis conditions for NH_2_-UIO-66 (Zr)@MIP, Figure S1: Diameter distribution of NH_2_-UIO-66 (Zr); Figure S2: EDS image of NH_2_-UIO-66 (Zr); Figure S3: Diameter distribution of NH_2_-UIO-66 (Zr)@MIP (a) and NH_2_-UIO-66 (Zr)@NIP (b); Figure S4: The high-angle annular dark-field (HAADF) image of NH_2_-UIO-66 (Zr)@MIP, and the corresponding element mappings of C, N, O and Zr; Figure S5: The FL response (*F*_0_/*F*) of NH_2_-UIO-66 (Zr)@MIP to different concentrations (2.5–40 μg mL^−1^) of OTC, TET, DOX, CTC and CAP, as well as to 40 μg mL^−1^ OTC + 40 μg mL^−1^ TET/DOX/CTC/CAP; Table S1: Optimization of the molar ratio of OTC, MAA and EGDMA; Table S2: Optimization of NH_2_-UIO-66 (Zr) addition; Table S3: Optimization of polymerization time; Table S4: The intra- and inter-day precisions of the designed FL assay (*n* = 6); Table S5: Comparison of the constructed NH_2_-UIO-66 (Zr)@MIP-based sensing approach with other reported FL methods for OTC detection in milk.
