# Supplementary material for: A Turn-Off Fluorescent Biomimetic Sensor Based on a Molecularly Imprinted Polymer-Coated Amino-Functionalized Zirconium (IV) Metal–Organic Framework for the Ultrasensitive and Selective Detection of Trace Oxytetracycline in Milk
Source: Foods. 2023 Jun 3;12(11):2255. doi: 10.3390/foods12112255 (PMC10252438; doi:10.3390/foods12112255)
Supplement: Supplementary file 1 [file foods-12-02255-s001.zip › foods-2432132-supplementary.pdf]

A turn-off fluorescent biomimetic sensor based on a molecularly imprinted polymer-coated amino-functionalized zirconium (IV) metal-organic framework for the ultrasensitive and selective detection of trace oxytetracycline in milk

Xiaohui Wang[1], Chang Liu[2], Yichuan Cao[1], Lin Cai[1], Haiyang Wang[1] and Guozhen Fang[1,]*

[1] State Key Laboratory of Food Nutrition and Safety, Tianjin University of Science and Technology, Tianjin 300457, China; xhw2022tjin@163.com (X.W.); cycydx2019@126.com (Y.C.); cailin511@163.com (L.C.); ocean5416@163.com (H.W.)

[2] School of Food Science, Henan Institute of Science and Technology, Xinxiang 453003, China; liuchang@hist.edu.cn (C.L.)

* Correspondence: fangguozhen@tust.edu.cn;

Tel.: +86-22-6091-2493;

Fax: +86-22-6091-2493.

Instruments

The morphology and size of synthetic materials were observed by scanning electron microscope (SEM, Hitachi, Japan) and transmission electron microscope (TEM, JEOL, Japan). Fourier transform infrared (FT-IR) spectra were collected using a Tensor-37 FT-IR spectrophotometer (Bruker, Germany) to investigate the functional groups of materials. X-ray diffraction (XRD) data were obtained by a D8 X-ray diffractometer (Bruker, Germany) in the 2θ range from 4° to 70° with Cu Kα radiation to analyze the crystal structure of as-prepared materials. Nitrogen adsorption-desorption analysis was carried out on an autosorb IQ instrument (Quantachrome, USA) and the specific surface areas were determined by the Brunauer-Emmett-Teller (BET) approach. Fluorescence spectra were measured on an F-7100 fluorescence spectrophotometer (Thermo, USA) with the excitation wavelength of 350 nm. UV-vis absorption spectra were acquired by an ultraviolet spectrophotometer (Victoria, Australia).

The detection conditions for HPLC method

The experimental parameters of HPLC detection system equipped with a Thermo Scientific-C18 analytical column (4.6 mm×250 mm, 5 mm) were described as below: The mobile phase was oxalic acid (0.01 mol L^{-1})/acetonitrile/methanol (77/18/5, v/v/v) with a flow-rate of 1.0 mL min^{-1}. The ultraviolet detector wavelength was 350 nm, and the column temperature was set at 40°C. The injected volume of each sample was 20 μL.

The sample analysis procedures for HPLC method

Briefly, 10 mL milk samples with various OTC concentrations (0 μg mL^{-1}, 1 μg mL^{-1},

15 μg mL^{-1} and 25 μg mL^{-1}) were added into the 50 mL centrifuge tubes. The target molecule OTC was extracted twice using 40 mL of Na_2EDTA-Mcllvaine buffer solution. After centrifugation, the twice supernatants of samples were combined and passed through a solid-phase extraction column that had been pre-treated with methanol and water. Then the column was rinsed with 5 mL of water/methanol mixture (v/v, 19:1). The target OTC was eluted with 5 mL of methanol and stored in a centrifuge tube. The above elution solution flowed through a carboxylic acid type cation exchange column. Thereafter, the exchange column was washed with 5 mL of methanol, drained under reduced pressure and eluted with 4 mL of oxalic acid-acetonitrile solution (v: v, 1:1). The collected solution was blown dry with nitrogen and re-dispersed in 1 mL of the mobile phase for subsequent HPLC detection.

Optimization of synthesis conditions for NH_2-UIO-66 (Zr)@MIP

In order to obtain polymers with desirable binding ability and excellent fluorescence response to OTC, the synthesis conditions including the molar ratio of OTC, MAA and EGDMA, the addition amount of NH_2-UIO-66 (Zr) and polymerization time were optimized. The imprinting factor (*IF*) was defined as the specific value of $K_{SV,\ NH_2\text{-UIO-66 (Zr)@MIP}}$ to $K_{SV,\ NH_2\text{-UIO-66 (Zr)@NIP}}$, and employed as a crucial indicator to appraise the comprehensive performance of the sensor.

To our knowledge, the appropriate proportion of OTC, MAA and EGDMA is conducive to the formation of molecularly imprinted polymer (MIP) with certain thickness and stable reticular structure, directly affecting the adsorption effect and fluorescence intensity of the polymers. According to the data in Table S1, when cross-

linking agent was insufficient, MIP_1 with network structure was not well formed, thereby reducing its adsorption capacity to OTC. However, excess cross-linking agent might thicken the layer of MIP_3, which not only weakened the sensitivity of fluorescence sensor, but also hindered the interaction between template molecules and NH_2-UIO-66 (Zr). Significantly, the maximum *IF* was acquired with the molar ratio of 1:6:10 (MIP_2), indicating that the sensor had the best recognition ability to the target OTC. Hence, 1:6:10 was used as the best molar ratio throughout the research.

The addition amount of NH_2-UIO-66 (Zr) as a meaningful fluorescence indicator parameter was studied to improve the fluorescence performance and sensitivity of the sensor. As revealed in Table S2, when the additive amount of NH_2-UIO-66 (Zr) was 50 mg, the sensor displayed the strongest fluorescence quenching response to OTC with the maximum *IF* value of 3.74. Therefore, 50 mg NH_2-UIO-66 (Zr) were added in the preparation procedure.

Furthermore, in the process of synthesizing MIP, a short reaction time makes it impossible for MIP to form a stable network structure, which affects the adsorption performance of the sensor. Whereas, the excessive increase of reaction time may lead to the high cross-linking of MIP, thus affecting the luminescence characteristics of the sensor. Thus, the polymerization time was optimized, and the results (Table S3) showed that the optimum reaction time was 15 h.

Table S1 Optimization of the molar ratio of OTC, MAA and EGDMA.

Polymer	Ratio	F_0/F (NH_2-UIO-66(Zr)@MIP)	F_0/F (NH_2-UIO-66(Zr)@NIP)	IF
MIP_1	1:6:8	2.551	1.399	3.89
MIP_2	1:6:10	2.812	1.455	3.98
MIP_3	1:6:20	2.437	1.407	3.53

Table S2 Optimization of NH_2-UIO-66 (Zr) addition.

Polymer	Dosage (mg)	F_0/F (NH_2-UIO-66(Zr)@MIP)	F_0/F (NH_2-UIO-66(Zr)@NIP)	IF
MIP_1	40	2.513	1.428	3.54
MIP_2	50	2.681	1.449	3.74
MIP_3	60	2.551	1.429	3.62

Table S3 Optimization of polymerization time.

Polymer	Time (h)	F_0/F (NH_2-UIO-66(Zr)@MIP)	F_0/F (NH_2-UIO-66(Zr)@NIP)	IF
MIP_1	10	2.576	1.401	3.93
MIP_2	15	2.703	1.415	4.11
MIP_3	20	2.241	1.380	3.26

Table S4 The intra- and inter-day precisions of the designed FL assay (n = 6).

Sample	Spiked ($\mu g\ mL^{-1}$)	Intra-day (n = 6)[a]			Inter-day (n = 6)[b]		
		Detected Concentration ($\mu g\ mL^{-1}$)		RSD (%, n = 6)	Detected Concentration ($\mu g\ mL^{-1}$)		RSD (%, n = 6)
Milk	1.00	0.97 ± 0.03		3.05	0.95 ± 0.04		4.07
	15.00	14.65 ± 0.11		0.73	14.57 ± 0.40		2.72
	25.00	24.34 ± 0.24		0.97	24.31 ± 0.40		1.63

[a] Intra-day precision: the average value of six repeated tests within the same day

[b] Inter-day precision: the average value of the same experiment on six different days

Table S5 Comparison of the constructed NH_2-UIO-66 (Zr)@MIP-based sensing approach with other reported FL methods for OTC detection in milk.

Fluorescent material	Linear range ($\mu g\ mL^{-1}$)	LOD ($ng\ mL^{-1}$)	Recovery (%)	RSD (%)	Reference
SiNPs	0.09-9.21	82.88	99.00-104.00	< 0.24	[52]
N-CDs@mMIPs	4.60-46.04	16.12	99.80-100.60	< 1.25	[53]
SiQDs	0.92-9.20	190.00	98.80-100.50	< 2.31	[31]
Cu NCs@PEI	0.23-138.13	14.73	99.80-104.90	< 3.29	[54]
NCDs/Eu^{3+}	0.05-11.51	13.35	94.40-96.00	< 4.90	[55]
CDs@HZIF-8	0.23-18.42	13.56	96.29-100.41	< 6.61	[56]
Mg,N-CDs@MIL-101@MIP	0.05-40.00	16.80	91.20-96.20	< 3.10	[37]
CDs	2.30-18.42	188.78	93.90-98.40	< 5.48	[29]
NH_2-UIO-66 (Zr)@MIP	0.05-40.00	12.28	93.56-98.21	< 2.59	This work

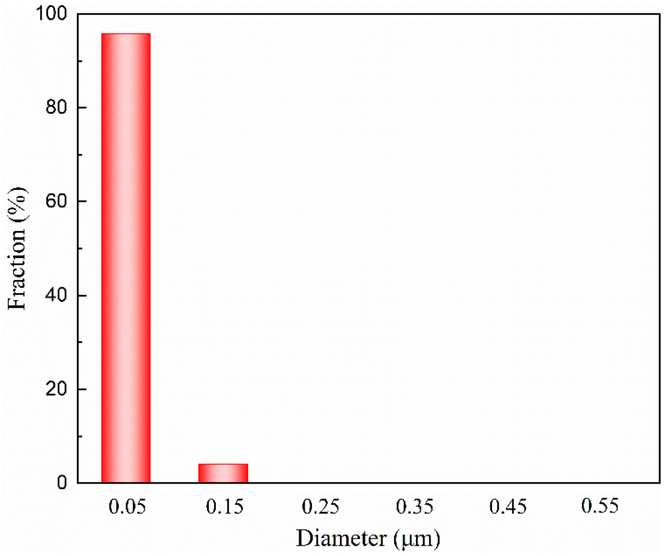

Fig.S1. Diameter distribution of NH_2-UIO-66 (Zr).

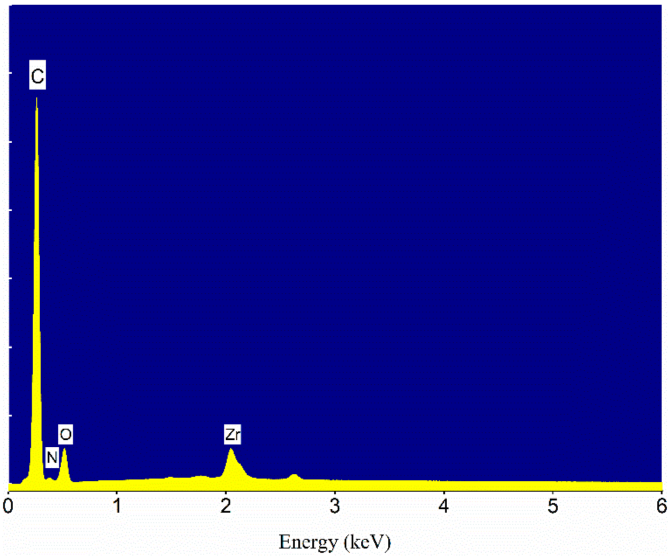

Fig.S2. EDS image of NH_2-UIO-66 (Zr).

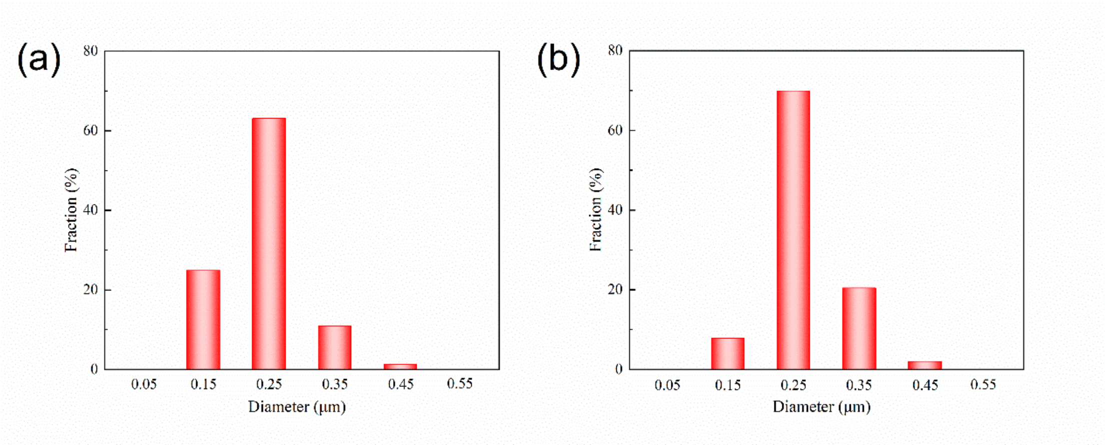

Fig.S3. Diameter distribution of NH_2-UIO-66 (Zr)@MIP (a) and NH_2-UIO-66 (Zr)@NIP (b).

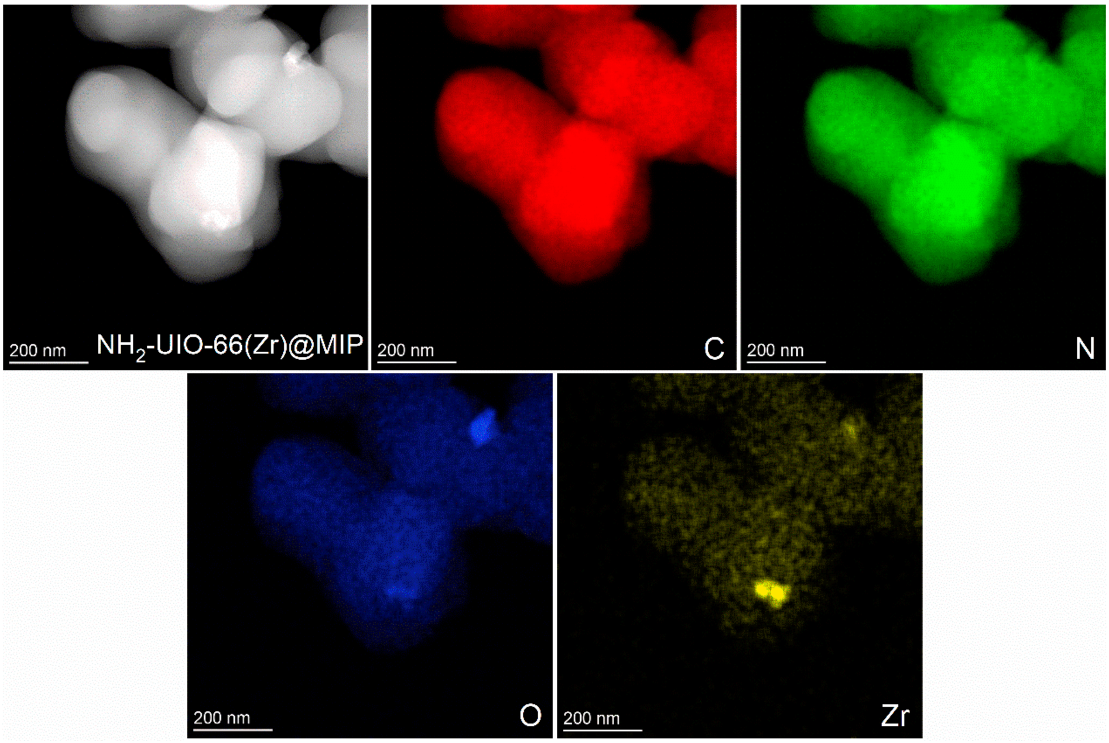

Fig. S4. The high-angle annular dark-field (HAADF) image of NH_2-UIO-66 (Zr)@MIP, and the corresponding element mappings of C, N, O and Zr.

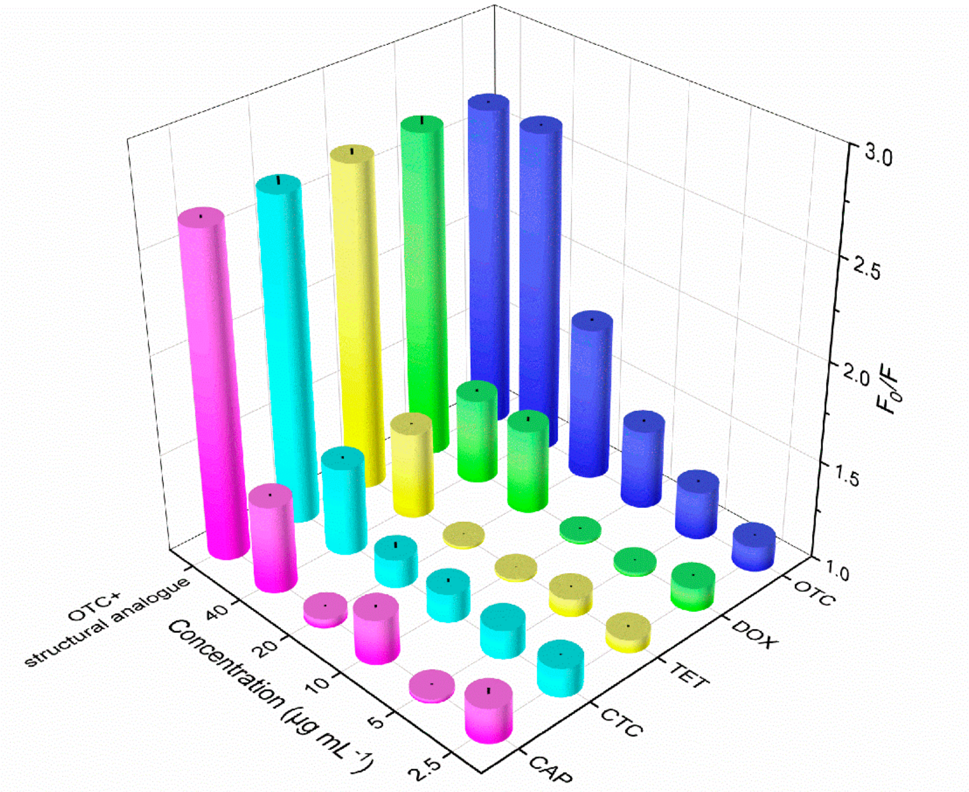

Fig. S5. The FL response (F_0/F) of NH_2-UIO-66 (Zr)@MIP to different concentrations (2.5-40 μg mL^{-1}) of OTC, TET, DOX, CTC and CAP, as well as to 40 μg mL^{-1} OTC+40 μg mL^{-1} TET/DOX/CTC/CAP.
